# Development and validation of the Attribution of Mental States Questionnaire (AMS-Q): A reference tool for assessing anthropomorphism

**DOI:** 10.3389/fpsyg.2023.999921

**Published:** 2023-02-16

**Authors:** Laura Miraglia, Giulia Peretti, Federico Manzi, Cinzia Di Dio, Davide Massaro, Antonella Marchetti

**Affiliations:** ^1^Research Unit on Theory of Mind, Department of Psychology, Università Cattolica del Sacro Cuore, Milan, Italy; ^2^Research Unit on Robopsychology in the Lifespan, Department of Psychology, Università Cattolica del Sacro Cuore, Milan, Italy

**Keywords:** attribution of mental states questionnaire, mentalization, mental states, factor analysis, validation, adults, theory of mind

## Abstract

Attributing mental states to others, such as feelings, beliefs, goals, desires, and attitudes, is an important interpersonal ability, necessary for adaptive relationships, which underlies the ability to mentalize. To evaluate the attribution of mental and sensory states, a new 23-item measure, the Attribution of Mental States Questionnaire (AMS-Q), has been developed. The present study aimed to investigate the dimensionality of the AMS-Q and its psychometric proprieties in two studies. Study 1 focused on the development of the questionnaire and its factorial structure in a sample of Italian adults (*N* = 378). Study 2 aimed to confirm the findings in a new sample (*N* = 271). Besides the AMS-Q, Study 2 included assessments of Theory of Mind (ToM), mentalization, and alexithymia. A Principal Components Analysis (PCA) and a Parallel Analysis (PA) of the data from Study 1 yielded three factors assessing mental states with positive or neutral valence (AMS-NP), mental states with negative valence (AMS-N), and sensory states (AMS-S). These showed satisfactory reliability indexes. AMS-Q’s whole-scale internal consistency was excellent. Multigroup Confirmatory Factor Analysis (CFA) further confirmed the three-factor structure. The AMS-Q subscales also showed a consistent pattern of correlation with associated constructs in the theoretically predicted ways, relating positively to ToM and mentalization and negatively to alexithymia. Thus, the questionnaire is considered suitable to be easily administered and sensitive for assessing the attribution of mental and sensory states to humans. The AMS-Q can also be administered with stimuli of nonhuman agents (e.g., animals, inanimate things, and even God); this allows the level of mental anthropomorphization of other agents to be assessed using the human as a term of comparison, providing important hints in the perception of nonhuman entities as more or less mentalistic compared to human beings, and identifying what factors are required for the attribution of human mental traits to nonhuman agents, further helping to delineate the perception of others’ minds.

## 1. Introduction

The ability to mentalize (Fonagy, 1989, 1991; Fonagy and Bateman, 2008; Fonagy and Luyten, 2009), also called Theory of Mind (ToM; Premack and Woodruff, 1978; Wimmer and Perner, 1983; Perner and Wimmer, 1985; Wellman et al., 2001; Wellman, 2020), is a human-specific ability that allows attributing mental states – intentions, thoughts, desires, and emotions – to themselves and others to explain and predict behavior (Gopnik and Wellman, 1992; Wellman, 1992; Frith and Frith, 1999, 2006; Tomasello, 1999; Astington and Baird, 2005; Tomasello et al., 2005). Mind reading abilities are a crucial function of social cognition that enables engagement in human interactions and promotes adaptation in everyday social contexts (Mull and Evans, 2010). In daily life, the ability to mentalize allows people to function socially by distinguishing between accidental and intentional behavior, desires and reality, truth and deception (Bellagamba et al., 2012), and to reach goals, including understanding, predicting, or controlling another’s behavior, as well as being able to understand the perspective of others, feel sympathy or compassion, and provide help (Davis et al., 1996; Batson et al., 1997; Galinsky et al., 2005; Waytz et al., 2012; Goldstein et al., 2014). So, nearly all children and adults consistently use their mind-reading skills for everyday social purposes. In this sense, the nature of social behaviors is rarely neutral and more often are behaviors that require prosocial or antisocial use of ToM skills (Ronald et al., 2005; Arefi, 2010). For these reasons, Ronald et al. (2005) proposed the expressions *nice Theory of Mind* and *nasty Theory of Mind* to distinguish prosocial and antisocial ToM abilities (Happé and Frith, 1996), identifying nice ToM in behaviors such as cooperating, comforting, considering others’ feelings, and nasty ToM, which involves an intact mentalizing ability but used to manipulate, outwit, or tease others (Happé and Frith, 1996).

Mentalization skills, necessary for children’s social functioning (Astington, 2003) and emotion regulation (Greenberg et al., 2017), develop from early dyadic relationships with mothers (Fonagy et al., 1991, 2007; Nelson, 2005; Slaughter et al., 2009; Fink et al., 2012; Meins et al., 2012), within which infants experience mental states through maternal language that contains references to the mental sphere (Beeghly et al., 1986; Symons et al., 2006; Slaughter et al., 2009; Giovanelli et al., 2020). However, words for mental states are not immediately understood by infants because of their abstract and invisible form (Slaughter et al., 2009). The development of mental states vocabulary begins at approximately 2 years of age within conversations with mothers who explicitly label children’s mental states for them (Bretherton and Beeghly, 1982; Bartsch and Wellman, 1995; Slaughter et al., 2009). In this regard, a large body of research reveals that mothers’ tendency to talk about emotions, desires, and beliefs and to make verbal references to their children’s mental experiences provides relevant input into children’s emerging mentalistic vocabulary (Meins et al., 2002; Nelson, 2005; Symons et al., 2006; Taumoepeau and Ruffman, 2006, 2008; Slaughter et al., 2009). Later, mothers’ tendency to make verbal references fades, and by age 4/6, children develop an awareness that others may have mental states different from their own (Wimmer and Perner, 1983). The development of a mentalistic vocabulary allows children to reflect on and understand their own and others’ mental states, assuming that the other is structurally endowed with a mind capable of possessing internal mental states. Thus, from childhood, the attribution of mental states to others becomes an ongoing process that occurs constantly and continuously throughout the lifespan to understand, explain, and reduce uncertainty about people’s behaviors. Importantly, we also make inferences about nonhuman agents’ internal states to approach and interact with them (Waytz et al., 2012; Martini et al., 2016; Di Dio et al., 2018), i.e., non-anthropomorphic living entities (e.g., animals), anthropomorphic non-living entities (e.g., robots), non-living non-anthropomorphic entities (e.g., objects), and even God (Heider and Simmel, 1944; Abell et al., 2000; Giménez-Dasí et al., 2005; Harris and Koenig, 2006; Ramsey and Hamilton, 2010; Gervais, 2013; Wigger et al., 2013; Wellman, 2017; Di Dio et al., 2018; Marchetti et al., 2018; Manzi et al., 2020b, 2021b,c). As noted by Waytz et al. (2010) perceived similarity between self and another individual increases as one considers their mental state. At the same time, the characteristics of an agent, animate or inanimate, influence the perception of its mind. For instance, dogs are ascribed special mental properties due to some of their species-specific sensory characteristics – e.g., the sense of smell that allows the perception of an object closed in a sealed box – that is much more developed than in humans (Di Dio et al., 2018). In addition, regardless of religious background, preschoolers attribute qualities such as omniscience to the mind of God, thus perceiving God’s mind at a higher epistemic level than humans’ minds (Nyhof and Johnson, 2017; Di Dio et al., 2018). Several studies have focused also on the attribution of minds to robotic agents (for a review, see Thellman et al., 2022) and observed that adults are more inclined to ascribe greater mental states to robots characterized by human-like physical features (Dario et al., 2001; MacDorman et al., 2005; Kiesler et al., 2008; Krach et al., 2008; Bartneck et al., 2009; Fink et al., 2012; Gray and Wegner, 2012; Airenti, 2015; Złotowski et al., 2015; Thellman et al., 2017; Wiese and Weis, 2020; Manzi et al., 2020b, 2021a,b,c). This tendency has also been found in children over the age of five, who are likely to attribute more mental states to robots with more human-like features; in contrast, younger children tend to anthropomorphize by giving less importance to the human aspect of the robotic agent (Di Dio et al., 2020a,b; Manzi et al., 2020b). Attributing mental states and consequently perceiving an agent as more or less mentalistic has important implications on how one will interact with it because mind perception implies moral status (Gray et al., 2007; Waytz et al., 2010). In fact, ascribing mind has consequences for both the perceiver and the perceived (Waytz et al., 2010), to the point of making it relevant to evaluate the perception of the minds of different entities as compared to humans.

The present study aimed to validate a new and agile measure, the Attribution of Mental State Questionnaire (AMS-Q) – already widely used in studies with children (Di Dio et al., 2018, 2020a,b; Manzi et al., 2020b; Peretti et al., 2023) and adults (Manzi et al., 2021c) – which assesses the attribution of mental and sensory states primarily to human. However, to the authors’ knowledge, there is no currently validated measure to compare the mental traits of human and nonhuman agents to evaluate the level of mental anthropomorphization of nonhuman agents, including living and nonliving entities. The AMS-Q aims to fill this void, as its originality lies in comparing the attribution of mental states between human and nonhuman agents by also administering pictures of nonhuman agents as stimuli. In this sense, the human picture is used as a baseline to assess, through comparison, the level of mental anthropomorphization of nonhuman agents (e.g., animals, inanimate things, and even God). The general purpose of the current study was to validate the AMS-Q on human stimuli across two Italian samples and then to show its sensitivity in capturing differences in the attribution of mental and sensory traits between human and nonhuman agents through an example of the applicability of the questionnaire in which an image of a dog and a robot were administered as nonhuman agent stimuli in addition to the human baseline. This research consisted of two main studies preceded by a preliminary study aimed to develop and generate an initial item pool based on the previous version of the AMS-Q (Manzi et al., 2017, 2020; Di Dio et al., 2018) and on a wide corpus of literature. Study 1 investigated the structure of the questionnaire, whereas Study 2 focused on confirming the factor structure and aimed to investigate the construct validity of the questionnaire by investigating its convergent and divergent validity. The rationale, design, and hypotheses of each study are outlined in more detail in the following sessions.

### 1.1. Study hypotheses

Congruent with theoretical formulations postulating that people intuitively think about others’ minds in distinct dimensional representations (Gray et al., 2007; Malle, 2019), we expected at least a two-factor model of the AMS-Q, with scales that distinctly assessed mental states attribution and sensory states attribution. We investigated the reliability and validity of the AMS-Q in two samples of Italian adults. Exploratory Factor Analysis and (multi-group) Confirmatory Factor Analysis (CFA) were used to investigate the factor structure of the questionnaire. Two different groups were recruited for the exploratory (*N* = 378) and confirmatory (*N* = 271) analysis.

Research Unit on Theory of Mind, Department of Psychology, Università Cattolica del Sacro Cuore, Milan, Italy.

The convergent validity of the AMS-Q was investigated by administering the Reading the Mind in the Eyes test (ET; Baron-Cohen et al., 2001; Italian version: Vellante et al., 2013), an advanced Theory of Mind test to evaluate the correspondence between the semantic definition of mental state and the image of the eye-region displayed on the screen. Differing from other measures that assess the individual’s mental abilities, the Eye Test explicitly evaluates the ability to *attribute* mental states to others. However, the ET assesses mental states predominantly related to the emotional sphere. To overcome this limitation, we also included a second measure: the Multidimensional Mentalizing Questionnaire (MMQ; Gori et al., 2021), which aims to assess core aspects of mentalization, including the cognitive sphere. The MMQ, in fact, is a self-report measure, which assesses mentalization on four central axes (cognitive-affective, self-other, outside-inside, and explicit-implicit). We expected the AMS-Q subscales to be significantly positively correlated with the ET and the MMQ. We also correlated the AMS-Q and the Toronto Alexithymia Scale (TAS-20; Italian version: Bressi et al., 1996), to test for divergent validity. TAS-20 is a self-report scale designed to evaluate the level of alexithymia, i.e., the inability to describe and/or distinguish one’s own emotions (Westwood et al., 2017; for a detailed description of scales, see Methods: Measures section). Negative correlations between AMS-Q and alexithymia were expected, as this dimension indicates poor awareness of emotions and feelings and mind-blindness.

Finally, the discriminant validity of AMS was investigated by testing its ability to differentiate between the attribution of mental and sensory states toward different entities. For this purpose, in addition to images of humans, we administrated two other stimuli: a picture of a robot (non-living entity) and a dog (living non-human entity). We assumed that the AMS-Q would be able to capture differences in terms of attributions of mental and sensory states between the human agent and the other two entities, allowing us to assess the level of mental anthropomorphism attributed to the agents examined.

## 2. Scale development: Item generation

Several sources were used in generating the initial item pool: the psychological lexicon of Lecce and Pagnin (2007); Slaughter et al.’ (2009) theoretical model of mental verb categorization resulting from communicative exchanges between mother and child; and Martini et al. (2016) work. The initial item pool also included the mentalistic verbs of the earlier version of the AMS scale, which has been widely used in research with children (Di Dio et al., 2018, 2019; see also, Manzi et al., 2017, 2020; Di Dio et al., 2020a,b) and adults (Manzi et al., 2021c).

Mentalistic vocabulary has been selected to encompass different categories of mental states: (a) volition (i.e., nouns, verbs, adjectives, or adverbs referring to states of desire or intention); (b) cognition (i.e., nouns, verbs, adjectives, or adverbs referring to mental acts of thought, intellect, or reasoning); and (c) disposition (nouns, verbs, adjectives, or adverbs referring to states of preference or affect; Slaughter et al., 2009). We also included a category referring to sensory states (e.g., smell, listen, look, taste, etc.) in the initial item pool. The resulting 69 mentalistic verbs and expressions were administered to fifty (50) Italian speakers, aged 18+ years (48.7% females; *Mean age* = 35.36; *SD* = 13.89). Participants were recruited through a mailing list built by the research team over time. Included in the email to the participants was an invitation letter and a link to access the online task on the Qualtrics platform. All participants participated on a voluntary and anonymous basis. They received no compensation for participating in the study.

To produce a valid factorial analysis, we asked participants to choose five words for each mental verbs category, after having looked at pictures depicting specific human characters (i.e., “Select five words/expressions that, according to you, are most representative to describe the image”). Each participant looked at five stylized, black-and-white images of human beings administered in random order: a woman, a man, a girl, a boy, and an infant.

Descriptive frequency analysis revealed the mentalistic expressions or verbs most selected by participants. This word evaluation method was chosen to provide a holistic approach to assessing the attribution of mental states in order to refine the items and to provide a questionnaire that can be representative of the concept of mental and sensory states related to human beings. Subsequently, the 26-item questionnaire was administered to a convenient sample of 22 (14 women and 8 men) Italian adults to investigate comprehensibility. This sample provided feedback on the clarity of item content and instructions, as well as on the images used. Items that were deemed odd or ambiguous were considered for rephrasing or exclusion. We decided to leave out two items that were defined as highly vague. The questionnaire was finally reduced to 24 items. In addition, overly detailed images of humans were discarded in favor of two black silhouettes because, especially the facial features drawn, seemed to suggest a state of mind that might could influence the attribution of emotional states.

## 3. Methods

### 3.1. Participants

The construction sample (Study 1) included 378 Italian adults (54.2% female; *Mean age* = 30.6; *SD* = 12.23; age-range = 18–65 years). Sociodemographic characteristics of the construction sample are reported in Table 1. The validation sample (Study 2) included 271 Italian adults (55.4% female; *Mean age* = 26.1, *SD* = 8.09; age-range = 19–60 years). Sociodemographic characteristics of the validation sample are reported in Table 1.

**Table 1:** Sociodemographic characteristics of the construction and validation samples.

**Sociodemographic characteristics**	**Construction sample** *N* = 378	**Validation sample** *N* = 271
Age, mean ± SD	30.6 ± 12.23	26.1 ± 8.09
Gender	N (%)	N (%)
Male	173 (45.8%)	121 (44.6%)
Female	205 (54.2%)	150 (55.4%)
Residence	N (%)	N (%)
North Italy	236 (62.6%)	222 (81.9%)
Centre Italy	64 (17%)	17 (6.3%)
South Italy	52 (13.8%)	20 (7.4%)
Sicily and Sardinia	25 (6.6%)	8 (3.0%)
Outside Italy		4 (1.5%)
Educational level	N (%)	N (%)
Middle school or below	9 (2.4%)	2 (0.8%)
High school	171 (45.2%)	169 (62.4%)
Graduate school	176 (46.5%)	95 (35%)
Postgraduate school	22 (5.8%)	5 (1.8%)
Employment status	N (%)	N (%)
Student	175 (46.3%)	171 (63.1%)
Employed	140 (37%)	73 (27%)
Unemployed	24 (6.3%)	4 (1.5%)
Other	39 (10.3%)	23 (8.5%)

All the participants were recruited on Prolific platform and rewarded with 6.35£ per hour. Written informed consent was obtained from all participants after a full explanation of the study procedure, in line with the Declaration of Helsinki and its revisions. The local Ethics Committee of the Department of Psychology, Università Cattolica del Sacro Cuore, Milan, approved the experimental protocol of all studies involved in the current research.

### 3.2. Procedure

Data were collected through an online survey hosted on the Qualtrics platform from November 2021 to January 2022.

**Figure 1:**
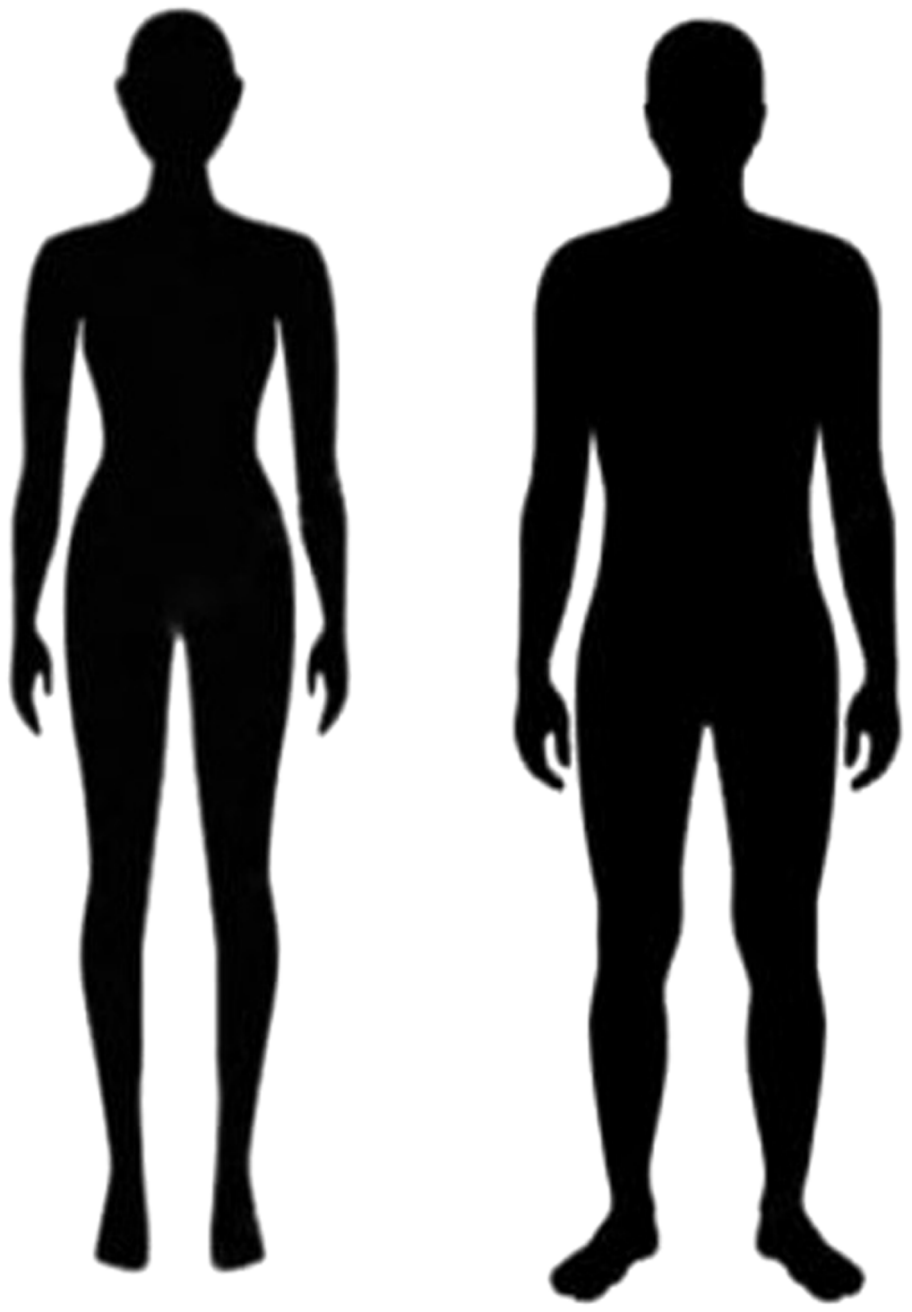
Stimuli for Study 1: silhouettes of a woman and a man.

With respect to Study 1, after the participants provided some sociodemographic information (age, gender, residence, occupation, and level of study), they completed the first version of the Attribution of Mental States Questionnaire in response to a male and female silhouette image evocative of human mentalistic traits (Figure 1). The items were randomized to avoid possible response bias by question order.

**Figure 2:**
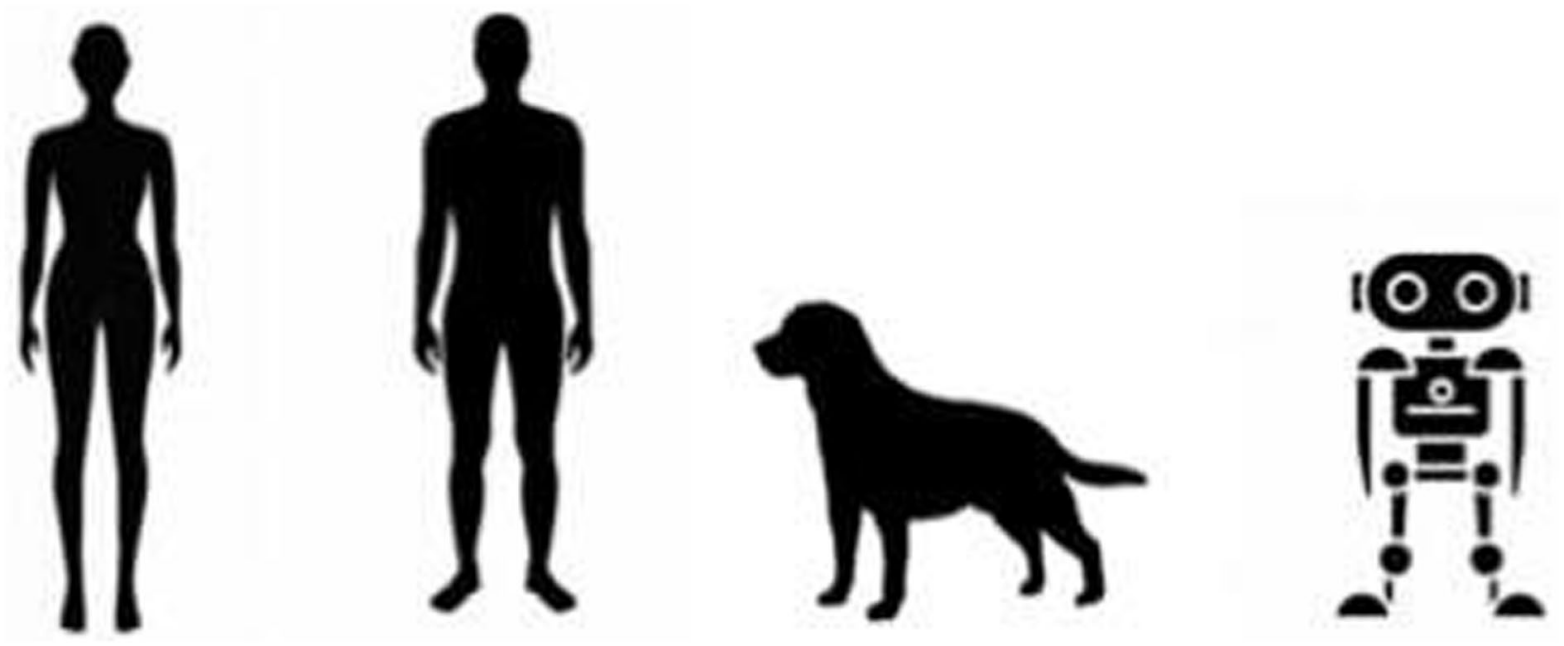
Stimuli for Study 2: silhouettes of a woman, a man, a dog, and a robot.

With respect to Study 2, participants completed a sociodemographic survey and the refined version of the AMS-Q in response to the male or female human silhouette. Participants completed the AMS-Q two more times with a robot and a dog picture as stimuli. The stimuli (Figure 2) were presented in random order. Finally, to test external validity, we correlated the questionnaires with validated tasks of Theory of Mind, mentalization ability, and alexithymia. All items were randomized to avoid participants’ responses may be affected by question order.

### 3.3. Measures

All the participants in the construction sample were administered a sociodemographic questionnaire assessing age, sex, residence, school attendance, current job, and the pool of 24 items composing the AMS-Q developed in the previous steps. Participants were asked to rate each item according to a 5-point Likert scale ranging from 1 (*No, not at all*) to 5 (*Yes, very much*). Participants were informed that they would have had to evaluate one of the two silhouettes’ images of human beings (i.e., “According to you, can a human being [*mental/sensory ability,* e.g.*, think/taste*]?”).

All the participants in the validation sample were administered the sociodemographic survey and a battery of questionnaires including the 24-item AMS-Q, the Multidimensional Mentalizing Questionnaire (MMQ), and the Italian version of the Reading the Mind in the Eyes Test (ET), and the Toronto Alexithymia Scale (TAS-20).

#### 3.3.1. Reading the Mind in the Eyes Test

The Reading the Mind in the Eyes Test (ET; Baron-Cohen et al., 2001; Italian version: Vellante et al., 2013) was administered to measure Theory of Mind and the attribution of mental states. Participants were randomly presented with a series of 36 photographs of the eye region of 19 actors and 17 actresses. Each photo was surrounded by four single-word mental state descriptors, e.g., bored, angry, happy. One of these descriptors targeted the mental state depicted in the photo, and the others were foils. The ET is based on a four-alternative forced-choice paradigm, with 25% correct guess rate. Participants were instructed to choose which of the four descriptors best describes what the person in the photo is thinking or feeling. The score on the test is the number of descriptors correctly identified by the participants, i.e., the number of mental states correctly identified. The maximum score is 36. In the validation sample, the internal reliability was acceptable (α = 0.52). As reported Vellante et al. (2013), there is some agreement that Cronbach’s coefficient alpha is a poor index of unidimensionality, in fact, the reliability of the Eyes test was rarely reported in past studies or obtained only acceptable values (Voracek and Dressler, 2006; Harkness et al., 2010).

#### 3.3.2. Multidimensional Mentalizing Questionnaire

Multidimensional Mentalizing Questionnaire (MMQ; Gori et al., 2021) is a self-report measure that consists of 33 items, covering the different core aspects of mentalization on four different axes: (1) cognitive-affective; (2) self-other; (3) outside-inside; and (4) explicit-implicit. It permits a multidimensional assessment, with scores on the positive (reflexivity, ego-strength, and relational attunement) and negative (relational discomfort, distrust, and emotional dyscontrol) subscales, as well as an overall MMQ score, by summing all the items after having reversed those included in the negative subscales. The response format was on a five-point Likert scale from 1 (not at all) to 5 (a great deal). In the current study, internal reliability was good (α = 0.80).

#### 3.3.3. Toronto Alexithymia Scale (TAS-20)

Toronto Alexithymia Scale (TAS-20; Italian version: Bressi et al., 1996) is a self-report scale comprising 20 items rated on a five-point scale ranging from 1 (strongly disagree) to 5 (strongly agree). It includes three subscales that measure three main dimensions of alexithymia: (1) difficulty in identifying feelings and distinguishing between feelings and bodily sensations in emotional activation, (2) difficulty in the verbal expression of emotions, and (3) externally oriented thinking. Taking the reversed items into account, the scores of the three scales were calculated. Internal reliability in the validation sample was good (α = 0.83).

### 3.4. Data analysis

#### 3.4.1. Study 1

In order to determine the dimensionality of the scale and sort out unsuitable items, we carried out an explanatory factor analysis using IMB SPSS Statistics version 27 and Jamovi statistical software version 2.5. A Principal Components Analysis (PCA) and a Parallel Analysis (PA; Horn, 1965) were carried out on the 24-item. PA is an adaptation of the Kaiser criterion eigenvalue >1 (Kaiser, 1960), and minimizes the tendency to identify a greater number of factors due to sampling error. PA uses the 95th percentile of the distribution of eigenvalues generated from uncorrelated data and, therefore the number of factors extracted is considered to be “beyond chance.”

Prior to performing PCA, the adequacy of the correlation matrix for factor analysis was assessed with Bartlett’s test of sphericity and the Kaiser-Meyer-Olkin (KMO) test. Adequacy of the correlation matrix is suggested by a significant Bartlett’s test (*p* < 0.05) and a KMO index >0.70. To examine the factor structure that underpins the AMS questionnaire, the PCA was carried out *via* oblique rotation (Promax) as the factors were presumably related to each other rather than independent. Delta was set to 0. Only items with a loading ≥0.30 (Hair et al., 1998) on a single factor were considered for further analyses. The solution revealed through PCA was further supported by the results of the PA.

Then, we investigated the internal consistency of the questionnaire and the presence of problematic items (i.e., items for which the Cronbach alpha improved). No items were removed and the version of the questionnaire with all 24 items was selected as it reported excellent reliability (α > 0.95).

#### 3.4.2. Study 2

The factor structure of the AMS-Q was subjected to a Confirmatory Factor Analysis (CFA) to confirm the three-factor model revealed in Study 1. To perform the analyses, Jamovi statistical software version 2.5 was used. Multi-group CFA was carried out using JASP team (2020). In order to evaluate the goodness-of-fit of the factor structure, we used the χ^2^/*df* ratio. A model in which χ2/*df* is ≤3, is considered acceptable. Furthermore, Hu and Bentler (1999) guidelines for fit indices were used to determine whether the expected model fitted the data. The following fit indices were used: (a) the Comparative Fit Index (CFI), with values ≥0.90 indicating a good fit (Bentler, 1990; Fan et al., 1999; Hu and Bentler, 1999); (b) the Tucker Lewis Index (TLI), with values ≥0.90 indicating a reasonable fit of the model (Byrne, 1994); (c) the Root Mean Square Error of Approximation (RMSEA), with values between 0.05 and 0.08 indicating the adequacy of the model (Browne and Cudeck, 1993), and values ≤0.05 indicating evidence of absolute fit (Lai and Green, 2016); and (d) the Standardized Root Mean Square Residual (SRMR), with values ≤0.08 indicating an adequate fit (Hu and Bentler, 1999; Schermelleh-Engel et al., 2003).

Moreover, a multigroup CFA was performed to test invariance across gender of the final factor structure. We tested for configural invariance to assess whether the same number of factors is extracted across groups.

The validity of AMS-Q was assessed by correlating (Pearson *r*) the AMS-Q factors with theoretically related measures, namely the ET and the MMQ subscales to establish construct (convergent) validity. Second, we repeated the correlations between AMS-Q and TAS-20, to examine the discriminatory power of the measure and divergent validity.

Finally, to assess the discriminant validity of the AMS-Q we administered a picture of a living non-human agent (a dog) and a non-living non-human agent (a robot) in addition to the human stimuli. A repeated-measures GLM analysis comparing AMS-Q scores on human – i.e., the baseline –, dog, and robot stimuli was conducted to investigate the ability of the AMS-Q to discriminate between the attribution of mental and sensory states toward different entities. Comparison between the baseline and the two stimuli examined allows us to assess the level of mental anthropomorphism attributed to the dog and the robot. Greenhouse–Geisser correction for violations of the Mauchly sphericity test, *p* < 0.05, was used in the GLM analysis. All *post-hoc* comparisons were Bonferroni corrected.

## 4. Results

### 4.1. Study 1

#### 4.1.1. Exploratory factor analyses

**Figure 3:**
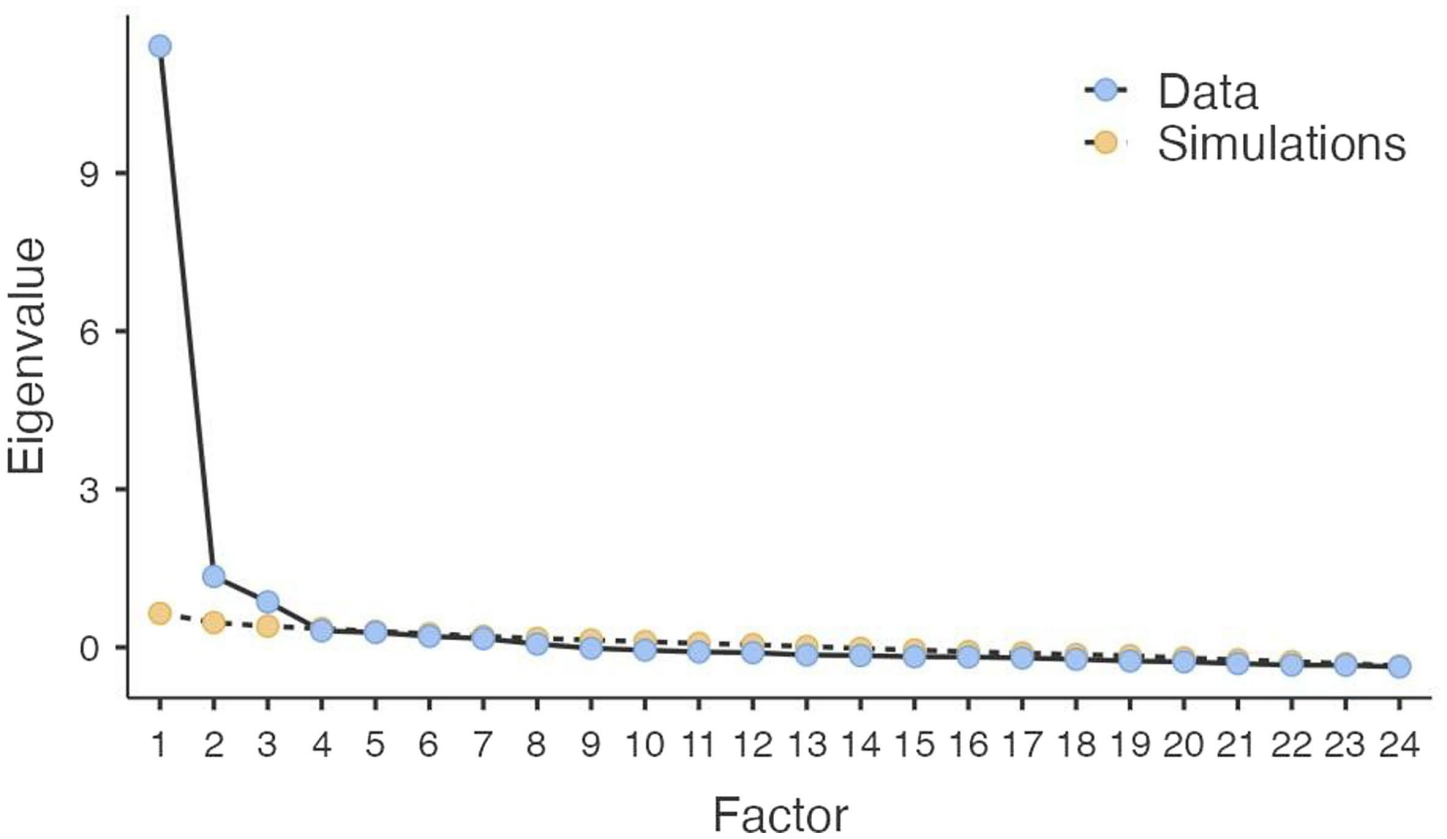
Scree plot: Eigenvalues for study 1 factor analysis.

A Principal Components Analysis (PCA) was carried out to explore the factors structure of the 24 items. The correlation matrix was suited for factor analysis (Bartlett’s test of sphericity = 6320.2, *df* = 276, *p* = 0.000; KMO = 0.95). The PCA yielded three components with eigenvalues over 1, explaining 49.7, 7.8, and 5.9% of the variance, respectively. Altogether, the extracted factors explained 63.4% of the total variance. Since Parallel Analysis (PA) is the most accurate method for component extraction (Zwick and Velicer, 1986; Hubbard and Allen, 1987), we proceeded by carrying out a PA on AMS data to confirm the structure previously found. The results of the analysis showed three components with eigenvalues exceeding the corresponding criterion values for a randomly generated data matrix of the same size (24 variables × 378 respondents). Thus, the questionnaire structure obtained from the PCA was confirmed by the results of the PA (Table 2). The inspection of the scree plot (Figure 3) also revealed that the three-factor solution was the most appropriate.

**Table 2:** Comparison of eigenvalues from PCA and criterion values from parallel analysis.

Factor	Actual eigenvalue from PCA	Criterion value from PA	Decision
F1: AMS-NP	11.926	11.409	Accept
F2: AMS-N	1.880	1.344	Accept
F3: AMS-S	1.409	0.862	Accept

The first extracted factor had 13 items with rotated loadings ranging from 0.32 to 0.83 (>0.30; Hair et al., 1998), assessing the attribution of knowledge states (beliefs, thoughts, inferences) and non-epistemic mental states such as planning, feelings, and positive emotions such as joy; consequently, it was labeled “Mental states with neutral or positive valence” (AMS-NP). The second extracted factor had seven items concerning the semantic field of deception (lying, pretending, making a joke) and related emotions with negative valence such as sadness, fear, and anger, which loaded strongly (between 0.59 and 0.91) on Factor 2. This factor can be named “Mental states with negative valence” (AMS-N). Finally, Factor 3 was composed of four items clearly associated with the attribution of sensory states with strong loadings between 0.69 and 0.91, which was accordingly called “Sensory States” (AMS-S). Individual item loadings on the retained components and the Cronbach’s alphas for each factor are listed in Table 3.

**Table 3:** Study 1 pattern matrix presenting loading factors for each item, percent of explained variance, and Cronbach’s alphas for each factor of the final factors.

**AMS items**	**Factor 1 AMS-NP**	**Factor 2 AMS-N**	**Factor 3 AMS-S**
Learn	0.728		
Think	0.747		
Remember	0.547		
Make a decision	0.609		
Understand	0.811		
Tell a lie		0.856	
Dream	0.535		
Imagine	0.829		
Make a joke		0.593	
Pretend		0.741	
See			0.828
Feel hot or cold		0.743	
Taste			0.638
Hear			0.910
Smell			0.857
Have fun	0.569		
Love	0.779		
Be happy	0.798		
Be sad		0.885	
Be scared		0.910	
Get angry		0.851	
Have the intention to do something	0.702		
Want to do something	0.668		
Make a wish	0.320		
% of explained variance	49.69%	7.84%	5.87%
Cronbach’s alpha	0.93	0.92	0.88

Extraction method: Principal Component Analysis. Rotation method: Promax with Kaiser Normalization. Rotation converged in 6 iterations.

#### 4.1.2. Reliability

The AMS-Q had excellent internal consistency, with a Cronbach alpha coefficient of 0.95. Partial alpha coefficients indicated that the three-component solution had satisfactory internal consistency (Factor 1 α = 0.93; Factor 2 α = 0.92; and Factor 3 α = 0.88). There was no relevant change (neither diminishment nor improvement) in overall reliability if any of the items were deleted.

### 4.2. Study 2

#### 4.2.1. Confirmatory factor analysis

Confirmatory Factor Analysis (CFA) was conducted on the three-factor model. First, we checked Bartlett’s sphericity test to ensure inter-item correlation (χ^2^ 3258.86, *df* = 325, *p* = 0.000) and the Kaiser–Meyer–Olkin (KMO = 0.93) for the sample adequacy.

Although the three-factor solution fitted the data well (χ^2^/*df* = 2.27; CFI = 0.89; TLI = 0.87; SRMR = 0.06; RMSEA = 0.07 [CI] = 0.061–0.076), coefficient R^2^ was suboptimal (R^2^ of 0.17) for item no. 2 (i.e., “*think*”), suggesting that the item’s variance was poorly represented by the common factor. However, for the promising indices reported in Table 4 and because the item is representative of attribution that would otherwise be lost, we decided not to remove it. Nevertheless, we decided to remove item no. 12 (“*feeling hot or cold*”) as it loaded moderately on two factors: respondents may possibly perceive this item as either a sensory state or a discomfort condition. Dropping out item no. 12 would then maximize the quality of responses.

**Table 4:** Goodness-of-fit indices generated by the Confirmatory Factor Analysis (CFA) with and without modification indices.

	Recommended value	Value obtained without MI	Value obtained with MI
χ2/*df*	≤ 3.00	2.27	1.87
CFI	≥ 0.90	0.89	0.93
TLI	≥ 0.90	0.87	0.92
SRMR	≤ 0.08	0.063	0.056
RMSEA	≤ 0.08	0.069 ([CI] = 0.061–0.076)	0.057 ([CI] = 0.048–0.065)

**Figure 4:**
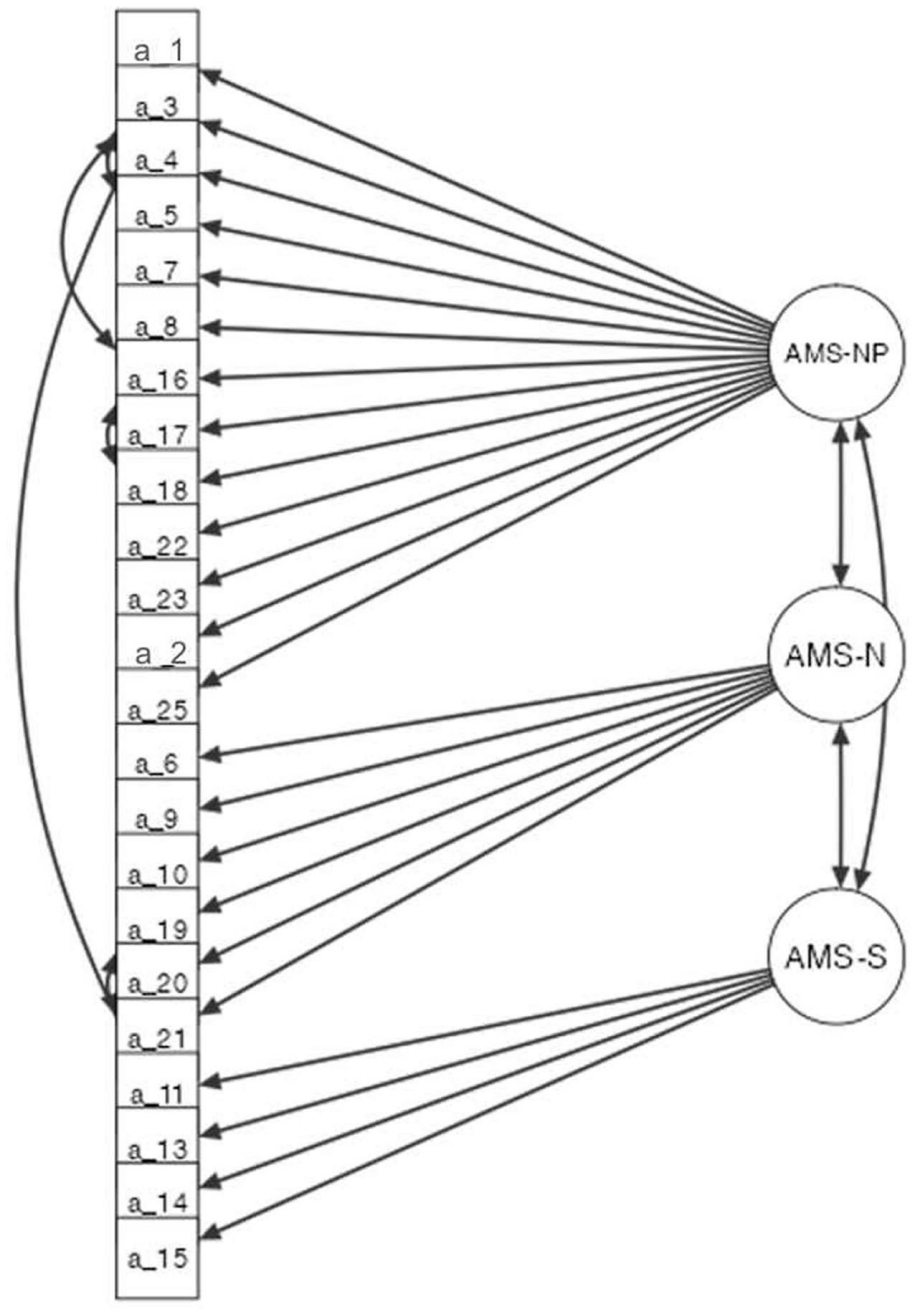
Graphical summary of the CFA obtained from the 23-item of the Attribution of Mental States (AMS-Q; N = 271).

Although most indices reached the recommended cut-off values (SRMR = 0.06; RMSEA = 0.07), the model could be improved, since inspection of modification indices (MI) >10 suggested that correlations between the errors of some pairs of items should be included in the model. CFA was re-run, and the goodness-of-fit indices indicated a satisfactory fit of the three-factor model. Indices with and without correlations between items are given in Table 4 (see also Figure 4).

The final version of the AMS-Q and the scoring is given in Appendix 1 and Appendix 2 (see Supplementary Material).

#### 4.2.2. Factor structure across gender

Next, to investigate the efficacy of the model across gender, separate multi-group CFAs were carried out for women (*N* = 150) and men (*N* = 121). The CFA on the refined and fully unconstrained model indicated an adequate fit (see Table 5), suggesting factorial invariance across gender. The indexes were in line with the recommended cut-off values.

**Table 5:** Goodness-of-fit indices generated by the multigroup CFA across gender.

	Recommended value	Value obtained
χ2/*df*	≤ 3.00	1.87
CFI	≥ 0.90	0.87
TLI	≥ 0.90	0.85
SRMR	≤ 0.08	0.08
RMSEA	≤ 0.08	0.08

#### 4.2.3. Correlations

Table 6 lists correlations of the AMS-Q subscales with convergent and divergent measures. The validity of the AMS-Q was tested through Pearson correlations with theoretically related measures, namely the ET and the MMQ to test convergent validity, and the TAS-20 to test divergent validity. As shown, AMS-Q subscales correlated significantly and in the expected direction with the ET, MMQ, and TAS-20:

**Table 6:** Pearson’s correlations between measures.

	AMS-NP	AMS-N	AMS-S
ET	**0.171** **	**0.179** **	**0.154** *
MMQ-F1	**0.247** **	**0.323** **	**0.203** **
MMQ-F2	**0.164** **	0.104	**0.154** *
MMQ-F3	**0.246** **	**0.177** **	**0.183** **
TAS-DDF	**−0.138** *	−0.042	−0.099
TAS-DIF	**−0.135** *	0.000	−0.082
TAS-EOT	**−0.216** **	**−0.120** *	−0.105

* *p* < 0.05.

** *p* < 0.01.

N = 271. ET = Reading the Mind in the Eyes Test. MMQ = Multidimensional Mentalizing Questionnaire: MMQ-F1 = reflexivity; MMQ-F2 = ego-strength; MMQ-F3 = relational attunement. TAS-20 = Toronto Alexithymia Scale: TAS-DDF = difficulty identifying feelings; TAS-DIF = difficulty describing feelings; TAS-EOT = external oriented thinking. Significant correlations are in boldface.

#### 4.2.4. Convergent validity

All AMS-Q factors correlated significantly and in the hypothesized direction with the Eyes-test, *r* (AMS-NP) = 0.17, *p* < 0.01; *r* (AMS-N) = 0.18, *p* < 0.01; *r* (AMS-S) = 0.15, *p* < 0.05. Thus, the AMS-Q dimensions were correlated with convergent measures of ToM, configuring the AMS-Q as a questionnaire capable of assessing the attribution of mental states. Consistent with expectations, AMS-Q subscales correlated positively with measures of mentalizing abilities: Reflexivity scale of MMQ, *r* (AMS-NP) = 0.25, *p* < 0.01; *r* (AMS-N) = 0.32, *p* < 0.01; *r* (AMS-S) = 0.20, *p* < 0.01, and Relational Attunement scale of MMQ, *r* (AMS-NP) = 0.25, *p* < 0.01; *r* (AMS-N) = 0.18, *p* < 0.01; *r* (AMS-S) = 0.18 *p* < 0.01. AMS-NP and AMS-S correlated positively with the Ego-strength dimension of the MMQ, *r* (AMS-NP) = 0.16, *p* < 0.01; *r* (AMS-S) = 0.15, *p* < 0.05. As expected, no significant correlations were found with the other three factors of the MMQ – namely Relational Discomfort, Distrust, and Emotional Dyscontrol, *p* > 0.05 – as they refer to failures and distortions of mentalization abilities that are reflected in relationships and interpersonal difficulties, which are dimensions that AMS-Q does not evaluate.

#### 4.2.5. Divergent validity

AMS-Q subscales were inversely correlated with the TAS, as expected. In particular, AMS-NP negatively correlated with Difficulty Identifying Feelings scale, *r* =. −14, *p* < 0.05, and Difficulty Describing Feelings scale of the TAS-20, *r* =. −13, *p* < 0.05. AMS-NP and AMS-N correlated negatively with External Oriented Thoughts, *r* =. −22, *p* < 0.01; *r* =. −12, *p* < 0.05.

#### 4.2.6. Discriminant validity

**Figure 5:**
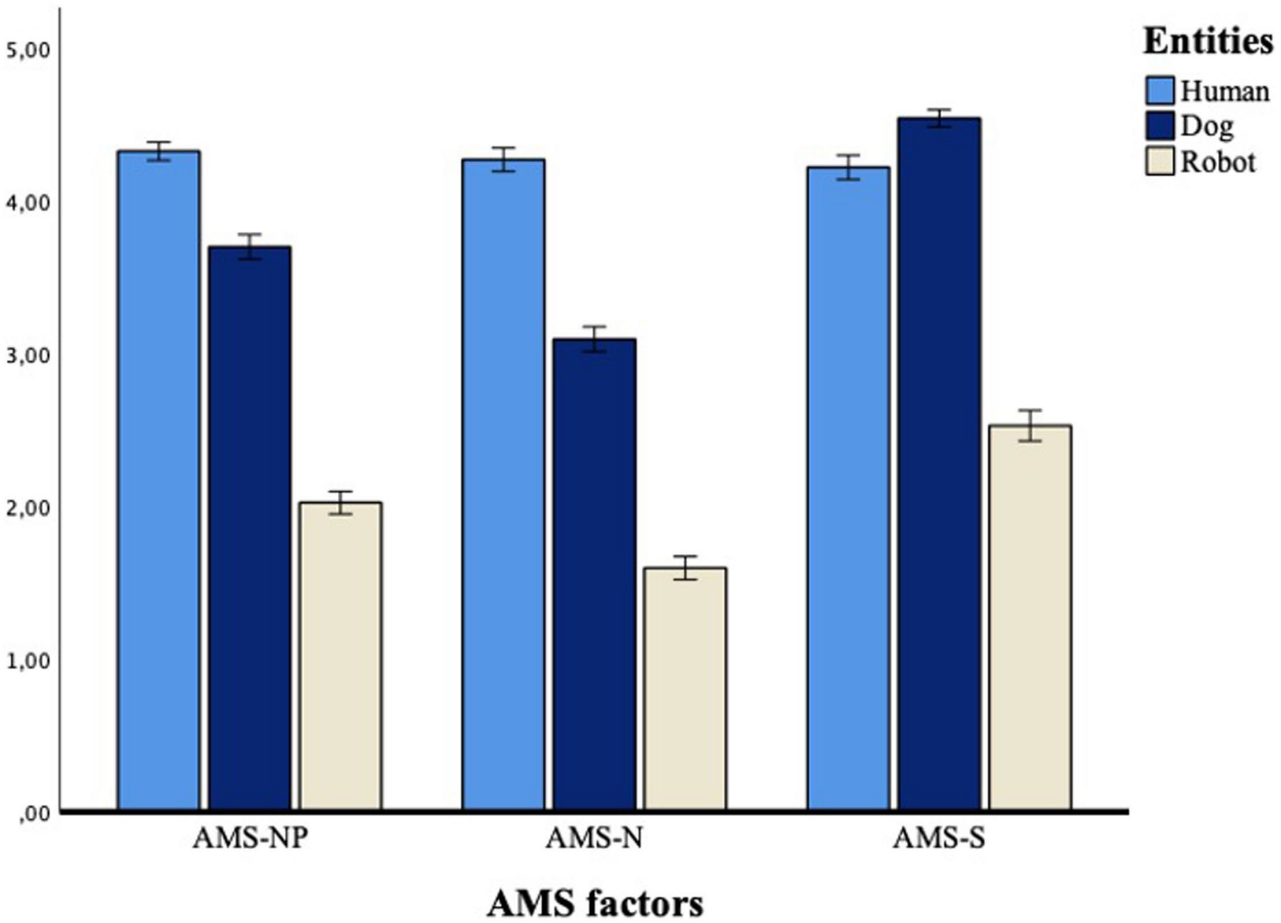
Differences among stimuli in the attribution of mental and sensory states.

The GLM analysis with three levels of *AMS-Q factors* (AMS-NP, AMS-N, AMS-S) and three levels of *entity* (human, dog, robot) as within-subjects factors, was conducted to evaluate the impact of different stimuli on participants’ scores on the AMS-Q. A main effect was found for the entity, *F*(1.68, 1315.85) = 1949.58, *p* < 0.001, partial-η^2^ = 0.89, δ = 1, indicating differences in participants’ mental states attribution toward the three different entities. Specifically, *post hoc* comparisons (Bonferroni corrected) showed participants’ tendency to ascribe greater mental states to the human than both the dog, M*diff* = 0.49, *SE* = 0.03, *p* < 0.001, and the robot, M*diff* = 2.22, *SE* = 0.04, *p* < 0.001. The dog also scored higher than the robot, M*diff =* 1.72, *SE* = 0.04, *p* < 0.001. The results also revealed a main effect of the interaction between entity and AMS-Q factors (Figure 5), *F*(3.27, 49.03) = 221.32*, p* < 0.001, partial-η^2^ = 0.45, δ = 1, indicating that humans scored higher on the attribution of knowledge states and positive emotions (AMS-NP) compared with both the dog, M*diff = 0*.63*, SE* = 0.04, *p* < 0.001, and to the robot, M*diff =* 2.30*, SE* = 0.05, *p* < 0.001. Respondents still attributed more negative value mental states (AMS-N) to humans than to dog, M*diff =* 1.18, *SE* = 0.04, *p* < 0.001, and robot, M*diff =* 2.67, *SE* = 0.05, *p* < 0.001. However, participants attributed greater positive (AMS-NP) and negative (AMS-N) value mental states to the dog compared to the robot, M*diff =* 1.67, *SE* = 0.05, *p* < 0.001; M*diff =* 1.50, *SE* = 0.05, *p* < 0.001. Finally, although more sensory states (AMS-S) were attributed to the human than to the robot, M*diff* = 1.69, *SE* = 0.06, *p* < 0.001; the dog was the highest scoring entity in attributing sensory states both compared to the robot, M*diff* = 2.01, *SE* = 0.05, *p* < 0.001, but also compared to the human M*diff* = 0.32, *SE* = 0.04, *p* < 0.001, pointing out the great sensitivity of the questionnaire to capture mental and sensory differences between different entities. Pairwise comparisons are listed in Table 7.

**Table 7:** AMS-Q differences in the attribution of mental and sensory states to a human, a dog, and a robot.

Entity	AMS-NP		AMS-N		AMS-S	
	M	*SD*	M	*SD*	M	*SD*
Human	**4.32**	0.03	**4.27**	0.04	4.22	0.04
Dog	3.70	0.04	3.09	0.04	**4.54**	0.04
Robot	2.02	0.04	1.60	0.04	2.53	0.05

The highest score for each AMS factor is bolded.

## 5. Discussion

The aim of the present study was to develop and validate a new questionnaire measuring the attribution of mental states to humans, the Attribution of Mental States Questionnaire (AMS-Q), across two Italian samples. In the current study, we aimed to provide a questionnaire validated with human stimuli that can be used as baseline for comparing the attribution of human mental and sensory traits to different entities – including living entities (e.g., animals, plants, etc.) and anthropomorphic and nonanthropomorphic nonliving entities (e.g., robots, objects, etc.) – and to assess the level of mental anthropomorphism attributed to them.

In Study 1, we found that a 24-item version of the questionnaire had excellent psychometric properties (α = 0.95) and a three-factor structure. Exploratory Factor Analysis revealed that Factor 1 (Mental states with neutral or positive valence – AMS-NP) is composed of 13 items: seven items concerning the attribution of epistemic mental states (beliefs, thoughts, inferences), three items concerning feelings, states of well-being, and positive emotions (love, have fun, and be happy), and three items concerning planning and volitional mental states (have the intention to do something, have a will to do something, and expressing a desire). Six items loaded on Factor 2 (Mental states with negative valence – AMS-N), three of which involved the attribution of cognitive mental states that belong to the semantic field of deception (i.e., tell a lie, deceive, and make a joke) and three related emotional states (be sad, angry, and afraid). Four items assessing the attribution of sensory states (i.e., hear, smell, look, and taste) are loaded on Factor 3 (Sensory states – AMS-S). This factor structure was confirmed in a new independent sample in Study 2 *via* Confirmatory Factor Analysis (CFA). Factors had high internal consistency and sufficient convergent and divergent validity. To further strengthen the structure of the questionnaire we revised the three-factor model by excluding one item (“*feeling hot or cold”*) and including correlations among errors for some pairs of items. The final version of the questionnaire included 23 items and maintained excellent internal consistency (α = 0.93). The abovementioned three-factor structure was also demonstrated through the multigroup CFA across gender. The goodness-of-fit indices were adequately close to support the three-factor model, which was also demonstrated by the strength of the factor loadings. This further means that females and males interpret the items in the same way and that the factor loadings are stable across groups.

As a reflection of this, the structure was also consistent from a theoretical perspective. The model revealed three factors that distinguished between the attribution of mental states (AMS-NP and AMS-N) and the attribution of sensory states (AMS-S). This was partially consistent with research showing that people intuitively think about other minds in terms of agency (the ability to plan and act) and experience (the ability to perceive and feel; Gray et al., 2007, 2011). The AMS questionnaire clearly distinguished the dual nature of the mentalistic lexicon, with mental states on one side and sensory states on the other. In Gray et al. (2007, 2011) the “experience” dimension of mental perception also includes the ability to feel fear, pain, pleasure, joy, etc. However, in the AMS questionnaire, these emotional states loaded into either the first or second factor (mental states). Also, in the present model, positive and negative emotions appeared to be at the opposite poles of a continuum of prosocial and antisocial use of mentalization ability. The former seems to be the emotional reactions resulting from prosocial behavior. On the other hand, negatively valenced emotions are loaded along with mentalistic verbs reflecting behaviors that require antisocial use of ToM abilities. Behaviors such as lying and deception fall under the concept of *Nasty ToM* (Happé and Frith, 1996) and are characterized by an intact but distorted mentalization ability in the domain of antisocial behavior (Ronald et al., 2005; McEwen et al., 2007; Lonigro et al., 2014). The fact that Factor 1 and Factor 2 items did not load on the same factor points to the possibility that mentalistic language distinguishes behaviors that on the value level are perceived as positive or neutral (e.g., thinking) or negative (e.g., pretending). Similarly, AMS-Q reflects real life, in which few social situations are neutral and the ability to grasp the intentions, beliefs, desires, and emotions of others can be used in prosocial or antisocial ways. Indeed, people consistently use their mind-reading abilities to understand and even control another’s behavior by manipulating, teasing, or other antisocial purposes (Arefi, 2010). Likewise, mentalizing abilities can offer help and cooperation, care about others, and consider their feelings. AMS-Q is thus able to capture the nuances of social behaviors that require the use of ToM, effectively distinguishing between “nice*”* and “nasty” ToM behaviors and their emotional consequences.

The AMS-Q demonstrated promising convergent validity as evidenced by correlations with validated measures of Theory of Mind and mentalization skills. The convergent validity of the AMS-Q was tested with the Eyes Test (ET; Baron-Cohen et al., 2001) which is considered to be an established measure of mentalization as it assesses adults’ ability to recognize the mental state of others using just the expressions around the eyes, which are key in determining mental states. The ET goes beyond merely assessing mentalizing abilities but assesses the extent to which people attribute mental states to others. This specificity made ET the ideal measure to correlate with AMS-Q because, although they have different purposes, both are based on the assessment of the attribution of mental states. As we expected, the AMS-Q subscales were significantly correlated with the ET, which means that the questionnaire is a valid tool that measures the attribution of mental states. Also, we found significant positive correlations with some scales of the Multidimensional Mentalizing Questionnaire (MMQ; Gori et al., 2021), a tool that assesses several core aspects of mentalization that, although all interrelated, concern relatively distinct capacities, such as cognitive-affective, self-other, outside-inside, and explicit-implicit. The Reflexivity, Ego-Strength, and Relational Attunement subscales refer to “positive” and functional components of mentalization (Gori et al., 2021) and are correlated with AMS factors as they focus on understanding others, acquiring their perspective, and being able to tune into the emotional and cognitive states of others and deeply understand their experiences. These are necessary components of mentalization and subsequent attribution of mental states. Conversely, we found no correlations with Relational Discomfort, Distrust, and Emotional Dyscontrol subscales since they refer to failures and distortions and evaluate manifestations of non-mentalizing states, which are not specifically assessed in the AMS-Q. Furthermore, as predicted, negative correlations were found between the AMS-NP and AMS-N and the construct of alexithymia; conversely, no correlations were found between the AMS-S and TAS-20 subscales. This is consistent as high scores in alexithymia indicate a difficulty in recognizing and attributing mental states; also, the AMS-S subscale assesses the ability to attribute sensory states while alexithymia can be defined as the inability to experience and identify emotions and reveals uncertainty about the emotional states of others and oneself.

In line with previous results with children and adults (Di Dio et al., 2018, 2020a,b; Manzi et al., 2020b, 2021c), the present data also showed that AMS-Q can discriminate the attribution of internal states to humans from nonhuman agents. In fact, the AMS-Q was able to differentiate between the entities used in the present study: human, dog, and robot. The GLM analysis indicated a significant difference in the attribution of mental and sensory states, resulting in greater attribution to humans than to robots and dogs, except for sensory states, where the dog was the highest-scoring entity. As a matter of fact, dogs have more developed senses (e.g., smell) than humans and this finding further enhances the sensitivity of the AMS to pick up on differences in the attribution of states, reflecting reality. Instead, the robot, contrary to the human and the dog, was perceived as an entity with low psychological and sensory skills. Overall, these results are in line with previous studies (Hackel et al., 2014; Martini et al., 2016; Di Dio et al., 2018, 2020a,b; Manzi et al., 2020b) reporting that different agents, or even the same agent with different characteristics (e.g., different types of robots; Manzi et al., 2020b), can evoke different – although diminished – attributions of human mental traits. Importantly, the tendency to attribute mental states to robots is also determined by factors such as people’s age, motivation, cultural background, and attitude toward robots, as well as the behavior, appearance, and identity of the robot (Marchesi et al., 2019; Thellman et al., 2022). Likewise, in a recent study, Manzi et al. (2021c) showed that humans are particularly sensitive to the design of robots in terms of attribution of mental qualities; in fact, even when robots differ slightly in their physical appearance, the dissimilar design evokes different mental properties. Consistently, previous studies in which the AMS-Q was administered (Di Dio et al., 2018, 2020, 2020a; Manzi et al., 2020a) have shown that children attribute qualitatively different internal states to humans compared to robots, highlighting the sensitivity of the AMS-Q in capturing these differences. Moreover, correlational studies with the AMS-Q have identified those factors, i.e., the age (Di Dio et al., 2020b; Manzi et al., 2020b) and the human likeness (for a review, see Marchetti et al., 2018) can influence the perception of the minds of robotic agents. In this framework, the AMS-Q stands as a valuable questionnaire that can capture the individuals’ ability to evaluate the level of mental anthropomorphism of nonhuman entities, including animals (Urquiza-Haas and Kotrschal, 2015), inanimate things (e.g., robot: Di Dio et al., 2020, 2020a; Manzi et al., 2020a, 2021c), paranormal entities (Gray et al., 2007), and even God (Di Dio et al., 2018); and provides interesting suggestions with respect to which factors may evoke different attributions of mental states. Therefore, the perception of the minds of living and nonliving beings has important implications. For instance, as Gray et al. (2007) have pointed out, there is a strong connection between the perception of mind and morality, such that attributing less mind to an entity also reduces its moral status, consequently affecting how people interact with that entity or agent. For example, the way people perceive and attribute mental states to others can lead to helping and praising or, conversely, denigrating and hurting. It may be concluded that the attribution of human mental traits (or the opposite dementalization) is predictive of attitudes (Urquiza-Haas and Kotrschal, 2015) and involve moral (Gray et al., 2007; Manzi et al., 2020a) and social evaluation processes (Kteily et al., 2016). Another advantage of the AMS-Q is that its data can be used flexibly in a variety of ways, as it allows for the investigation of the attribution of human mental states to nonhuman agents in order to assess the level of mental anthropomorphism. It may help explain the belief in God, the humanization of pets, and the attribution of responsibility to computers; and finally, it is a useful measure to identify which factors and conditions contribute to the increase or decrease in the process of mental anthropomorphizing. In conclusion, Dennett (1996) claimed that each mind is defined as such by the eye of the beholder, this is because it is individual perceptions that answer the question “what kind of things have a mind.” However, the AMS-Q has shown to be able to capture not only whether things have more or fewer minds but to explore their dimensions, capturing “nice*”* and “nasty” attributes and their emotional consequences.

## 6. Conclusion and limitations

Important conclusions can be drawn from the current study. The Attribution of Mental State Questionnaire (AMS-Q) has shown good psychometric properties; the rapid and easy administration of the measure allows a comprehensive assessment of the attribution of mental and sensory states to human and the comparison with nonhuman entities. Moreover, this research has highlighted the sensitivity of the AMS-Q in distinguishing between mental and sensory states, positive (or neutral) and nasty attributes and their emotional correlates, and in discriminating among agents in terms of mental states. The AMS-Q can be usefully adopted in research whose goal is to identify possible differences in the attribution of mental and sensory states between entities, using the human stimuli as baseline: the theoretical framework proposed here can provide important suggestions in the perception of nonhuman entities as more or less mentalistic comparable to humans. The AMS-Q may also provide insight into the possible difference between age groups and the factors required for human mental traits to be attributed to nonhuman agents, further helping to delineate the perception of others’ minds.

The study has some limitations that need to be acknowledged. Although the entire sample was of adequate size, there are significant differences in age, suggesting that the youngest may have greater weight in the analysis. In addition, our sample drew only from a nonclinical population.

It is worth noting a gender difference in levels of mentalization, with females having higher mentalization abilities than males (Focquaert et al., 2007; Dimitrijevic et al., 2017). This gender effect could affect anthropomorphic attribution and thus the outcome of the questionnaire. However, this bias does not seem to compromise the structure of the questionnaire presented in the article. This was also confirmed by the multigroup CFA: most indices were close to the recommended cutoff values. However, replication with larger samples would allow higher levels of certainty regarding the underlying three-factor structure.

Another limitation is to have used only two stimuli (dog and robot) to assess discriminant validity. However, our findings are supported by previous studies that indicate the sensitivity of AMS-Q to grasp differences in the attribution of mental and sensory states. It is important to note that the images we used were given as an example and were selected as representing the categories of living and non-living entities. AMS-Q is thought to be administered with a variety of stimuli, from animals to inanimate things, to paranormal entities, and even God. Thus, in future studies, stimuli different from those reported in this study can be administered, depending on the focus of the research question, always keeping human stimuli as baseline to assess the anthropomorphization of non-human agents.

Despite the above limitations, for the present time, the AMS-Q seems well-positioned to fill the void in mental states attribution measures and appears to have the potential as a reliable and psychometrically valid questionnaire for research applications, worthy of further empirical investigation. Although future research with AMS-Q involving different clinical samples and investigating structure stability over time is needed, the results of the studies reported in this article provide preliminary evidence for its reliability and validity and highlight possibilities for its broader application.

## Data availability statement

The raw data supporting the conclusions of this article will be made available by the authors, without undue reservation.

## Ethics statement

The studies involving human participants were reviewed and approved by Commissione Etica per la Ricerca in Psicologia, CERPS (Università Cattolica del Sacro Cuore, Milano). The patients/participants provided their written informed consent to participate in this study.

## Author contributions

All authors contributed to the study conception and design, commented on the initial versions, read, and approved the final manuscript. FM conceptualized the scale. CDD and LM secured ethical approval. LM, GP, and FM performed material preparation and data collection. LM carried out the statistical analysis. CDD suggested important improvements to the methodology. LM and GP wrote the first draft of the manuscript.

## Funding

This research was funded by Università Cattolica del Sacro Cuore (D.1).

## Conflict of interest

The authors declare that the research was conducted in the absence of any commercial or financial relationships that could be construed as a potential conflict of interest.

## Publisher’s note

All claims expressed in this article are solely those of the authors and do not necessarily represent those of their affiliated organizations, or those of the publisher, the editors and the reviewers. Any product that may be evaluated in this article, or claim that may be made by its manufacturer, is not guaranteed or endorsed by the publisher.
