# Supplementary material for: Development and validation of the Attribution of Mental States Questionnaire (AMS-Q): A reference tool for assessing anthropomorphism
Source: Front Psychol. 2023 Feb 16;14:999921. doi: 10.3389/fpsyg.2023.999921 (PMC9989770; doi:10.3389/fpsyg.2023.999921)
Supplement: Supplementary file 1 [file Presentation_1.pdf]

# *Appendix 1* – Attribution of Mental States Questionnaire (AMS-Q)

Answer the following questions using the scale provided: 1 No, not at all; 2 Yes, a little; 3 Yes, quite a bit; 4 Yes, a lot; 5 Yes, a lot.

| No, per nulla<br>*Not, at all* | 1 | 2 | 3 | 4 | 5 | Sì, moltissimo<br>*Yes, a lot* |
|---|---|---|---|---|---|---|

Secondo te, l'essere umano può […]?

*In your opinion, can human beings [...]?*

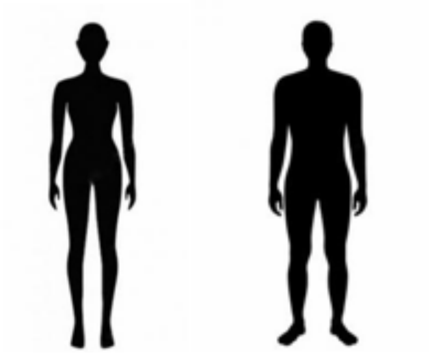

| AMS-NP | AMS-N | AMS-S | | | | | |
|---|---|---|---|---|---|---|---|
| 1. Imparare<br>*Learn* | | | 1 | 2 | 3 | 4 | 5 |
| 2. Pensare<br>*Think* | | | 1 | 2 | 3 | 4 | 5 |
| 3. Ricordare<br>*Remember* | | | 1 | 2 | 3 | 4 | 5 |
| 4. Decidere<br>*Make a decision* | | | 1 | 2 | 3 | 4 | 5 |
| 5. Capire<br>*Understand* | | | 1 | 2 | 3 | 4 | 5 |
| 6. Sognare<br>*Dream* | | | 1 | 2 | 3 | 4 | 5 |
| 7. Immaginare<br>*Imagine* | | | 1 | 2 | 3 | 4 | 5 |
| 8. Divertirsi<br>*Have fun* | | | 1 | 2 | 3 | 4 | 5 |
| 9. Voler bene<br>*Love* | | | 1 | 2 | 3 | 4 | 5 |
| 10. Essere felice<br>*Be happy* | | | 1 | 2 | 3 | 4 | 5 |
| 11. Avere intenzione di fare qualcosa | | | 1 | 2 | 3 | 4 | 5 |

| | | | | | | | |
|---|---|---|---|---|---|---|---|
| *Have the intention to do something* | | | | | | | |
| 12. Avere voglia di fare qualcosa<br>*Want to do something* | | | 1 | 2 | 3 | 4 | 5 |
| 13. Esprimere un desiderio<br>*Make a wish* | | | 1 | 2 | 3 | 4 | 5 |
| | 14. Dire una bugia<br>*Tell a lie* | | 1 | 2 | 3 | 4 | 5 |
| | 15. Fare uno scherzo<br>*Make a joke* | | 1 | 2 | 3 | 4 | 5 |
| | 16. Far finta<br>*Pretend* | | 1 | 2 | 3 | 4 | 5 |
| | 17. Essere triste<br>*Be sad* | | 1 | 2 | 3 | 4 | 5 |
| | 18. Avere paura<br>*Be scared* | | 1 | 2 | 3 | 4 | 5 |
| | 19. Arrabbiarsi<br>*Get angry* | | 1 | 2 | 3 | 4 | 5 |
| | | 20. Udire<br>*Hear* | 1 | 2 | 3 | 4 | 5 |
| | | 21. Annusare<br>*Smell* | 1 | 2 | 3 | 4 | 5 |
| | | 22. Guardare<br>*See* | 1 | 2 | 3 | 4 | 5 |
| | | 23. Gustare<br>*Taste* | 1 | 2 | 3 | 4 | 5 |
